# Non-uniform *in vivo* Expansion of Epstein-Barr Virus-Specific T-Cells Following Donor Lymphocyte Infusion for Post-transplant Lymphoproliferative Disease

**DOI:** 10.3389/fimmu.2019.02489

**Published:** 2019-10-29

**Authors:** David M. Burns, Gordon B. Ryan, Caroline M. Harvey, Eszter Nagy, Simon Hughes, Paul G. Murray, Nigel H. Russell, Christopher P. Fox, Heather M. Long

**Affiliations:** ^1^Institute for Immunology and Immunotherapy, University of Birmingham, Birmingham, United Kingdom; ^2^Cancer Immunology and Immunotherapy Centre, University of Birmingham, Birmingham, United Kingdom; ^3^Department of Clinical Haematology, Nottingham University Hospitals NHS Trust, Nottingham, United Kingdom; ^4^Department of Radiology, Nottingham University Hospitals NHS Trust, Nottingham, United Kingdom

**Keywords:** post-transplant lymphoproliferative disease, PTLD, Epstein-Barr virus, adoptive T-cell therapy, donor lymphocyte infusion, T-cells, flow cytometry, tetramers

## Abstract

Epstein-Barr virus (EBV)-associated post-transplant lymphoproliferative disease (PTLD) is a life-threatening complication of T-lymphocyte deplete allogeneic hematopoietic stem cell transplantation (allo-HSCT). For patients with PTLD refractory to Rituximab, donor lymphocyte infusion (DLI) is established as a successful option for salvage therapy. However, although *in vivo* lymphocyte expansion has been correlated with good clinical outcome following DLI, the specificity and functional characteristics of EBV-specific T-cell responses remain poorly characterized. Here we describe two patients with Rituximab-refractory PTLD complicating T-cell deplete allo-HSCT, both of whom were successfully rescued with 1 × 10^6^/Kg unselected stem cell donor-derived DLI. Prospective analyses revealed that complete clinical and radiological responses were associated with *in vivo* expansion of T and NK cells. Furthermore, EBV MHC tetramer, and interferon gamma analyses revealed a marked increase in EBV-specific T-cell frequency from 4 weeks after DLI. Reactivity was demonstrated against a range of EBV latent and lytic antigens, including those detected in tumor biopsy material. The immunodominant EBV-specific T cell response expanding *in vivo* following infusion matched the dominant response present in the DLI preparations prior to administration. Furthermore, differences in the repertoire of subdominant antigen-specific T-cells were also detected, suggesting that antigen-encounter *in vivo* can shape the immune response. These results demonstrate the value of prospectively studying *in vivo* T-cell responses, by facilitating the identification of important specificities required for clinical efficacy. Applying this approach on a larger scale promises to yield data which may be essential for the optimization of future adoptive immunotherapeutic strategies for PTLD.

## Introduction

Post-transplant lymphoproliferative disease (PTLD) remains a life-threatening complication of allogeneic hematopoietic stem cell transplantation (allo-HSCT) (1, 2). In this setting almost all cases arise from Epstein-Barr virus (EBV) transformed donor-derived B lymphocytes. EBV is carried as a persistent infection by more than 90% of the worldwide population (3). In healthy individuals, potent EBV-specific CD8+ and CD4+ T-cell responses against a range of viral antigens exert control over long-term infection (4). However, following allo-HSCT T-cell compromise may permit the opportunistic accumulation of infected B-cells, leading to PTLD. The reported incidence of disease after allo-HSCT, ranging from <1% to over 30% in some series, is therefore heavily influenced by factors related to host and graft T-cell suppression, in particular the use of T-cell depleting agents such as anti-thymocyte globulin (ATG) and Alemtuzumab (5).

Histologically, PTLD tumors exhibit a heterogeneous spectrum of pathologies but they are most commonly categorized as monomorphic diffuse large B-cell lymphomas (DLBCL) or polymorphic B-cell proliferations (6). In these, EBV genomes are detectable as a growth transforming infection, expressing a well-characterized set of latent virus gene products, comprising 6 EBV nuclear antigens (EBNA-1,-2,-3A,3B,-3C, and -LP), 3 latent membrane proteins (LMP-1,-2A,-2B) and BHRF1 (3). Several studies have also documented virus lytic cycle activity in a small fraction of tumor cells, a process driven by expression of the viral transcription factor BZLF1 (7, 8). However, some tumors, often those arising later after transplant, are less reliant on the EBV transforming genes and express a more limited range of viral genes, with concurrent cellular mutations.

Rituximab, a B lymphocyte-specific anti-CD20 monoclonal antibody, has significantly improved PTLD-related mortality following allo-HSCT (9), particularly when used as pre-emptive treatment in patients with raised circulating EBV DNA (10–13). Despite this, a proportion of patients fail to respond to, or relapse following, Rituximab therapy and are at high risk of mortality (9, 14). Whilst cytotoxic chemotherapy may be efficacious for Rituximab-refractory PTLD in the setting of solid organ transplantation, poor outcomes are reported after allo-HSCT, which is probably related to toxicity (14, 15). Considerably better clinical responses have been achieved using cellular therapies. Thus, transplant donor-derived unselected donor lymphocyte infusions (DLI), which contain EBV-specific T-cells whenever the donor is EBV-seropositive, have been used as successful salvage therapy for established PTLD, with response rates of around 70% (16–19). However, this approach is limited by the risk of alloreactive T-cell responses, which may result in potentially life-threatening graft-vs. -host disease (GvHD). Alternatively, EBV-specific cytotoxic T-cell (EBV CTL) infusions, prepared by *in vitro* stimulation of donor or third-party lymphocytes, avoid this complication. These have been used effectively both as prophylaxis and in the treatment of established disease, resulting in response rates similar to those achieved with DLI and, importantly, without evidence of alloreactivity (11, 20–22).

Unfortunately, EBV CTLs are still not universally available, due in part to the laborious and costly nature of their production (2). Novel approaches, including *ex vivo* selection of virus-specific T-cells (23–25) or genetically engineered T-cells (26), are seeking to address this issue. Clearly, the success of these selective adoptive cellular approaches will crucially depend on the targeting of appropriate antigens. It is notable therefore, that whilst *in vivo* expansion of adoptively transferred DLI and EBV CTLs has been correlated with successful clinical outcome (18), the dominant antigenic specificities present within polyclonal third party EBV CTLs prior to infusion do not correlate with clinical response (27), and thus the *in vivo* T-cell responses required to deliver clinical response are as yet poorly defined.

In the present study we propose that prospective analysis of the T-cell responses expanding *in vivo* after adoptive cell therapy might better shed light on the specificities required for effective therapeutic responses. As such we describe 2 cases of Rituximab-refractory EBV-positive PTLD arising after allo-HSCT successfully rescued using unselected DLI. We present the first detailed characterization of EBV epitope–specific T-cell responses both within the DLI, and within the *in vivo* expanded T-cells following infusion. We demonstrate non-uniform *in vivo* expansion of functional epitope-specific CD8+ and CD4+ T-cells recognizing viral antigens expressed within the PTLD tumor cells.

## Materials and Methods

### Patients

Both patients underwent allo-HSCT at Nottingham University Hospitals NHS Trust (NUH), Nottingham, UK, and were treated in accordance with institutionally approved protocols. The research was conducted with Research Ethics Committee and NHS Research and Development approval (12/WM/0147, West Midlands – Coventry and Warwickshire) and participants gave written informed consent in accordance with the Declaration of Helsinki. Patients undergoing allo-HSCT at NUH are routinely monitored with whole blood EBV qPCR testing weekly for at least 6 months post-transplant. Pre-emptive treatment comprising up to 4 infusions of Rituximab 375 mg/m^2^ is delivered to those exceeding an institutionally defined threshold of 10,000 EBV genomes/ml. PTLD was diagnosed in accordance with published criteria (28).

### Analysis of Lymphocyte Subsets

Peripheral blood mononuclear cells (PBMCs) were isolated using density centrifugation. PBMCs and aliquots of donor lymphocytes were analyzed immediately or cryopreserved. Thawed PBMCs were stained in MACS buffer on ice for 30 min using pre-determined concentrations of the following antibodies: CD14-Pacific Blue (M5E2), TCRα/β-AF488 (IP26), CD56-PE (HCD56), CD8-PerCP-Cy5.5 (SK1), and CD45-AF700 (HI30) from Biolegend; CD19-PE-Cy7 (HIB19), CD4-APC (SK3), and CD3-APC-eFluor780 (UCHT1) from eBioscience. After washing in MACS buffer, and addition of Sytox Blue (Invitrogen) for dead cell discrimination, cells were acquired on an LSRII (BD) flow cytometer (Beckman Coulter). Doublets, CD14+ monocytes and dead cells were excluded before a minimum of 30,000 CD45+ lymphocyte events were recorded. Data were analyzed using FacsDIVA software version 6.1.3 (BD). Lymphocyte counts, from routine clinical analysis of whole blood, were used to calculate lymphocyte subset frequency.

### EBV Epitope-Specific T-Cell Assays

Interferon-gamma (IFN-γ) release Elispot assays were performed on PBMC or donor lymphocytes stimulated at 2 × 10^5^ cells/well overnight using CD8+ and CD4+ T-cell EBV epitope peptides (Tables S1, S2) at a final concentration of 5 μg/ml, as previously described (29). All peptides were analyzed in duplicate or triplicate wells, and DMSO was used as a negative control. For MHC class I tetramer analysis, thawed PBMCs were exposed for 15 min at 37°C to pre-titrated volumes of APC-conjugated HLA A^*^0201 tetramers for EBV epitopes YVLDHLIVV (BRLF1 aa 109-117), GLCTLVAML (BMLF1 aa 280-288), and CLGGLLTMV (LMP2 aa 426-434) (30). The cells were subsequently washed in PBS, stained with LIVE/DEAD fixable violet dead cell stain (Life Technologies), and surface stained using pre-determined concentrations of CD3-AmCyan (SK7) and CD8-PE (RPA-T8) antibodies (BD Biosciences). After washing, the cells were acquired on an LSRII flow cytometer. Data were processed using FlowJo software version 7.6.5 (Tree Star). Absolute numbers of tetramer-specific T-cells were calculated from lymphocyte subset frequencies determined as described above.

### Immunohistochemistry and Epstein-Barr Virus-Encoded Small RNAs (EBERs) *in situ* Hybridization

For immunohistochemistry, sections from paraffin-embedded biopsy material were dewaxed in Histoclear for 10 min, rehydrated and quenched in 0.3% H_2_O_2_ for 15 min. The slides were subsequently boiled in citrate buffer pH6.0 for 20 min for antigen retrieval (40 min for BZLF1 staining). After blocking with Casein, the slides were incubated overnight at 4°C with primary antibodies diluted in PBS with 0.5% Tween20 against: EBNA1 IH4 (1:1,000), EBNA2 PE2 (1:2), LMP1 CS1-4 (Dako; 1:25), LMP2A 15F9 (Santa Cruz; 1:100), BZLF1 BZ.1 (neat), and gp350 72A1 (1:1,000). After 3 washes in PBS/Tween20, slides were incubated for 30 min at room temperature with the secondary antibody Dako Real EnVision HRP Rabbit/Mouse, or Dako Rabbit anti-Rat-HRP (1:100) for LMP2a staining. After three washes with PBS/Tween20, 100 μl diaminobenzidine (Dako) was applied to each slide for visualization, counter-staining with Meyers Hematoxylin. EBER *in situ* hybridization (EBER ISH) was performed with a Leica automated Bond system, using an EBER probe in combination with an anti-fluorescein antibody and Bond Polymer Refine Detection (Leica Biosystems), according to manufacturer's instructions.

## Results

### Patient Presentation and Treatment

This study used clinical data and samples collected from 2 patients who underwent treatment with DLI for Rituximab-refractory PTLD arising after allo-HSCT. Baseline and transplant characteristics for the patients are summarized in Table 1.

**Table 1:** Patient characteristics.

	**Patient A**	**Patient B**
Age at transplant, years	51	62
Sex	Female	Female
Diagnosis	LPL	AML
**FIRST TRANSPLANT**		
Donor	Unrelated	Sibling
Conditioning	Flu BEAM	Flu Mel
T-cell depletion	Alemtuzumab	Alemtuzumab
**SECOND TRANSPLANT**		
Donor	Unrelated	Sibling
Conditioning	Flu-Cy	FLAMSA-Bu
T-cell depletion	ATG	ATG
**EBV Serology**		
Recipient	+	+
Donor	+	+
**HLA Type**		
A	02, 32	01, 24
B	44, 49	07, 14
C	05, 07	07, 08
DRB1	01, 15	07, 15
DQB1	05, 06	02, 06

*LPL, lymphoplasmacytic lymphoma; AML, acute myeloid leukemia; Flu BEAM, Fludarabine, Carmustine, Etoposide, Cytarabine, Melphalan; Flu Mel, Fludarabine, Melphalan; Flu Cy, Fludarabine, Cyclophosphamide; FLAMSA-Bu, Fludarabine, Cytarabine, Amsacrine, ATG, Busulfan; ATG, antithymocyte globulin; EBV, Epstein-Barr virus; HLA, human leukocyte antigen; +, positive*.

**Figure 1:**
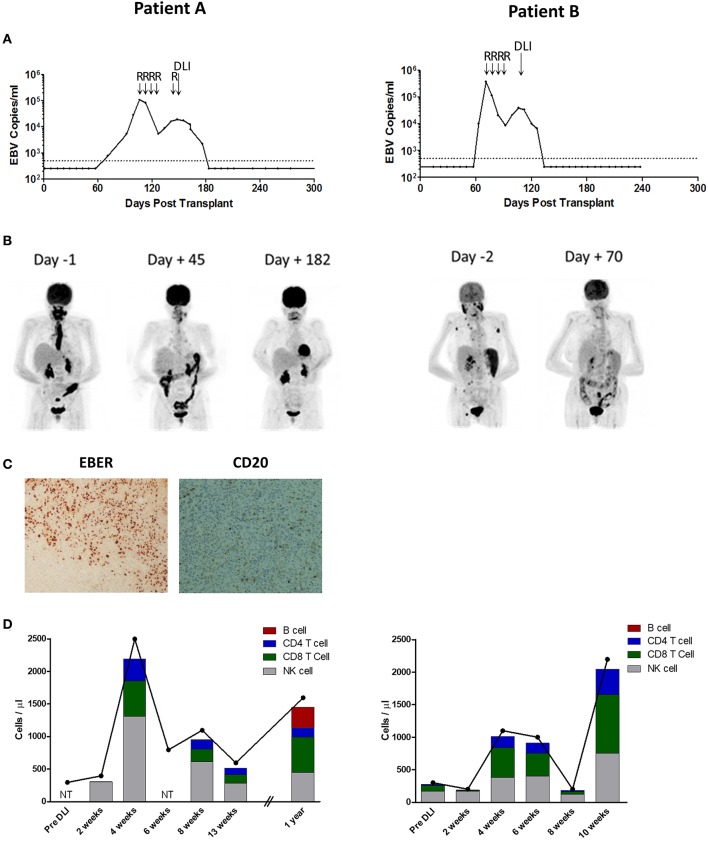
Patient **A** (Left) and **B** (Right) Investigations. **(A)** EBV loads in whole blood, measured by EBV qPCR, and shown as EBV copies/ml whole blood. Day 0 represents the time of second transplant. The dotted line represents the 500 copies/ml threshold of sensitivity for the EBV qPCR assay, below which values are arbitrarily shown as 250 copies/ml. R indicates infusion of Rituximab 375 mg/m^2^; DLI, donor lymphocyte infusion of 1.0 × 10^6^/Kg. **(B)** Positron emission tomography images taken pre and post-DLI infusion (Day 0). Patient A had active disease principally located in the oropharynx and esophagus. Patient B had active disease in the oropharynx, spleen and lymph nodes. **(C)** Analysis of formalin-fixed, paraffin-embedded diagnosis biopsy tissue sections from Patient A showing widespread positivity for EBERs on *in situ* hybridization but predominantly negative immunohistochemistry for CD20. Images are 40X magnified. **(D)** Kinetics of total lymphocyte (joined dots) and main lymphocyte subset counts (stacked columns), pre and post-DLI infusion. NT indicates not tested. Expansion of T-cell and NK-cell subsets was observed at 4 weeks after DLI in both patients.

Patient A underwent allo-HSCT for lymphoplasmacytic lymphoma in partial remission using Fludarabine, BEAM, and Alemtuzumab preparative conditioning, with an HLA-matched unrelated donor with a single HLA-A antigen mismatch (HLA A26 for A32). Due to graft failure, a second transplant from the same donor was performed using Fludarabine, Cyclophosphamide and ATG conditioning. EBV qPCR testing of whole blood revealed low-level EBV DNAemia on day 71 post second transplant, with a virus load of 760 copies/ml (Figure 1A, left). Despite weaning Cyclosporin, this subsequently rose to high-level EBV DNAemia (28,150 copies/ml) by day 99. The patient was treated with Rituximab 375 mg/m^2^, administered weekly to a total of 4 infusions, resulting in a decline in EBV load to a nadir of 5470 copies/ml on day 127. However, 7 days after the fourth infusion, there was recrudescence of EBV DNAemia which progressed on further testing. This was accompanied by severe oropharyngeal inflammation and ulceration causing absolute dysphagia and airway compromise which progressed despite empirical antimicrobial therapy and systemic steroids. Imaging including Positron emission tomography-computed tomography (PET-CT) revealed active disease principally involving Waldeyer's Ring and confluent, full-length esophageal involvement (Figure 1B, left). An oropharyngeal biopsy confirmed monomorphic PTLD of DLBCL subtype, which was positive for EBV by EBER *in situ* hybridization but negative for CD20 immunohistochemistry consistent with prior Rituximab exposure (Figure 1C) (31).

Patient B underwent allo-HSCT for AML in first complete remission, with Fludarabine, Melphalan, and Alemtuzumab preparative conditioning, using peripheral blood stem cells (PBSC) from a fully HLA-antigen matched sibling donor. A second allograft from the same donor was performed ~18 months later (day 0) following relapse of primary disease, with FLAMSA (ATG-containing) and Busulfan conditioning. EBV monitoring revealed a viral load of 9,810 copies/ml on day 63 (post second transplant) which progressed to 370,000 copies/ml on day 71 (Figure 1A, right). Consequently, pre-emptive therapy with Rituximab 375 mg/m^2^ was administered to a total of 4 weekly infusions, resulting in reduction of EBV load to a nadir of 8,625 copies/ml after the third infusion. However, 6 days following the fourth infusion there was recrudescence of EBV DNAemia with a virus load of 20,760 copies/ml which subsequently increased. This was associated with general malaise and B symptoms, and PET-CT revealed active disease involving the nasopharynx, spleen and multiple lymph nodes (Figure 1B, right). In the absence of an amenable biopsy site, a diagnosis of probable PTLD was made (28).

The patients were subsequently treated with single infusions of 1 × 10^6^/Kg unselected donor lymphocytes derived from their EBV-immune stem cell donors. Neither received chemotherapy. In both, a rapid and sustained response to DLI was observed, comprising reduction of EBV load to undetectable levels, resolution of symptoms (Figure 1A), and complete metabolic response defined by PET-CT (Figure 1B). Except for stage 1 skin GvHD in Patient A, administration of DLI was tolerated without complication. Patient A remains well after more than 3 years of follow-up. Patient B remained in remission from PTLD but died from relapsed AML around 8 months following their second transplant.

### Expansion of Lymphocyte Subsets Following DLI

Blood samples were collected prospectively following administration of DLI to monitor *in vivo* immune responses. Inspection of total lymphocyte counts revealed a similar pattern of lymphocyte expansion following DLI infusion in both patients (Figure 1D). Thus, marked lymphopenia was noted prior to infusion, with counts of only 300 cells/μl in both patients (normal range 1,000 – 4,000 cells/μl). Although these were unchanged at 2 weeks following infusion, by 4 weeks there were sharp increases to 2,500 cells/μl and 1,100 cells/μl for Patients A and B, respectively. Thereafter, the lymphocyte counts decreased to near baseline frequencies by 13 weeks in Patient A, and by 8 weeks in Patient B (although there was a further increase at the 10 week timepoint in the latter). In order to characterize lymphocyte subsets, PBMCs were analyzed using a multicolor flow cytometry panel to enumerate CD3+ T-cells (and CD8+ and CD4+ subsets), CD56+ natural killer (NK)-cells and CD19+ B-cells (Figure 1D). This revealed a predominance of NK-cells, and few T-cells, in the pre-DLI and 2 week timepoints. However, by 4 weeks both the CD8+ and CD4+ T-cell subsets underwent marked expansion, accompanied by a similar increase in NK-cells. Despite prior Rituximab therapy, CD19+ B-cells constituted 1.0% of lymphocytes at 2 weeks post-DLI-infusion in Patient A but reduced to 0.1% by the 4 week timepoint, and remained at trace levels at 13 weeks (maximum 0.3%) before increasing to 20.1% at 1 year. In Patient B, B-cells made up 1.6% of lymphocytes pre-DLI, falling to 0.6% at 2 weeks post-DLI and remained at trace levels at 10 weeks (maximum 0.2%). Overall, these data suggest that DLI-derived CD8+ and CD4+ T-cells and NK-cells all underwent *in vivo* expansion following infusion, co-incident with reduction in residual circulating B-cells.

### Characterization of EBV Epitope-Specific T-Cell Responses

Whilst previous studies have demonstrated global expansion of EBV-specific T-cells following administration of DLI, the antigenic specificities of these cells, which likely represent therapeutically important responses, have not been characterized (18, 32). To enumerate EBV epitope-specific T-cell responses for study patients, PBMCs were stimulated overnight with appropriate EBV peptides (detailed in Tables S1, S2) and responding CD8+ and CD4+ T-cells were quantified using IFN-γ release Elispot assay.

**Figure 2:**
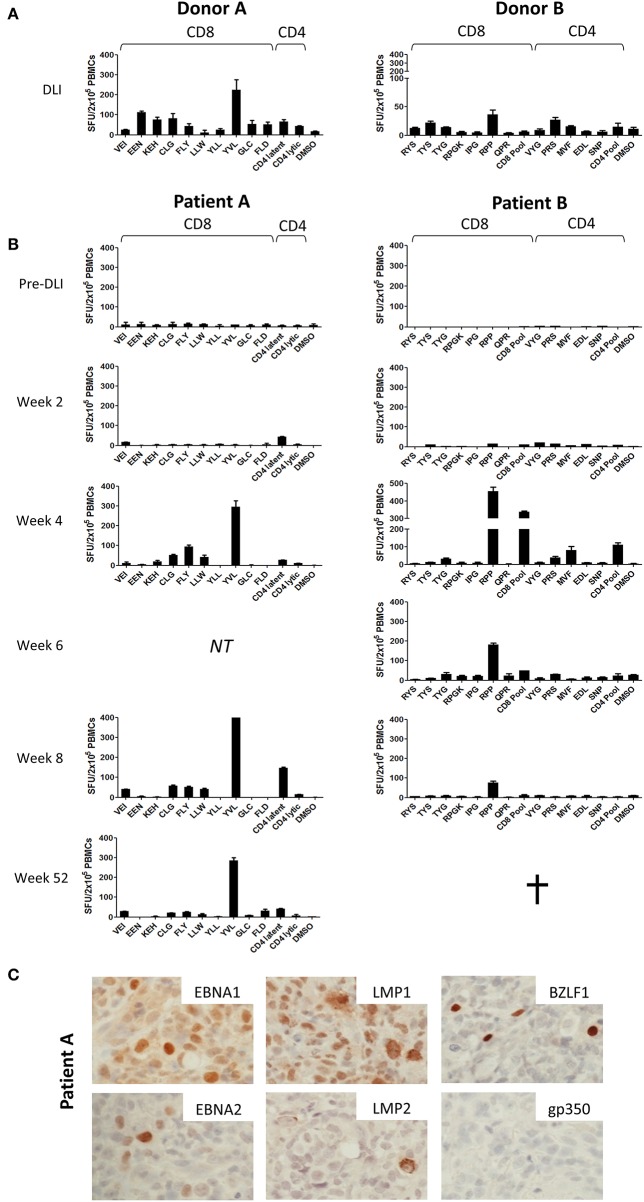
Prospective analysis of EBV epitope-specific T-cell responses. Aliquots of the infused DLI **(A)** or PBMCs from serially collected samples from patients A and B **(B)** were stimulated overnight with selected panels of CD8+ and CD4+ epitope peptides of relevant HLA class I and class II restriction (Tables S1, S2) before enumeration of responding cells using IFN-γ Elispot. Results shown are the mean spot forming units (SFUs) per 2 × 10^5^ cells from duplicate or triplicate wells +/– SD. **(C)** Immunohistochemistry analysis of Formalin-fixed, paraffin-embedded diagnosis biopsy sections from Patient A, showing strong positivity for latent EBV antigens EBNA1, EBNA2, LMP1, and LMP2 and the lytic EBV antigen BZLF1 but absence of the late lytic cycle antigen gp350. Images are 60X magnified.

Initially, we tested aliquots of the donor lymphocytes administered to each patient (Figure 2A). In both donors, EBV-specific CD8+, and CD4+ T-cell responses to a range of EBV antigens were observed, noting prominent responses to immunodominant epitopes, as typically seen in healthy EBV seropositive individuals (33–35). DLI given to Patient A (left panel) exhibited strong responses to the CD8+ T-cell epitopes EEN (EBNA3C), KEH (EBNA3C), CLG (LMP2), and YVL (BRLF1), whilst the DLI for Patient B (right panel) showed responses against the CD8+ T-cell peptides TYS (EBNA3B) and RPP (EBNA3A), and the CD4+ T-cell peptide PRS (EBNA2).

In contrast, analysis of patient PBMCs collected prior to DLI infusion showed responses undetectable above background (Figure 2B). Responses remained poor at 2 weeks after DLI. However, by 4 weeks there was a marked increase in EBV-specific T-cell frequency. For each case, emergent immunodominant T-cell responses matched those present at the highest frequency in the corresponding DLI samples. However, it was also notable that subdominant responses included some, but not all, of the epitope specificities identified in the DLI. Thus, in Patient A (left panel) the CD8+ response was dominated by reactivity against the CD8+ YVL (BRLF1) peptide, with weaker responses to the LMP2 peptides FLY, CLG, and LLW, and very low/undetectable responses to EEN (EBNA3C), GLC (BMLF1), and FLD (BALF4). In Patient B (right panel) the RPP (EBNA3A) peptide and the CD8+ peptide pool (a composite of lytic antigen peptides) elicited the strongest responses, whereas responses to RYS (EBNA3A) and TYS (EBNA3B) were almost undetectable. Epitope-specific CD4+ T-cells were also detected in both patients, most evidently against the pool of CD4+ latent epitope peptides in Patient A, and the MVF (EBNA1) peptide and CD4+ peptide pool (a composite of lytic antigens plus EBNA3C peptides) in Patient B. Interestingly, CD4+ T-cell responses may have preceded those of CD8+ T-cells in Patient A at week 2. The responses persisted in both patients, most notably Patient A in whom EBV-specific T-cells were detectable at both 52 weeks and 2 years following DLI (data not shown).

### Expression of EBV T-Cell Target Antigens in PTLD Tissue

**Figure 3:**
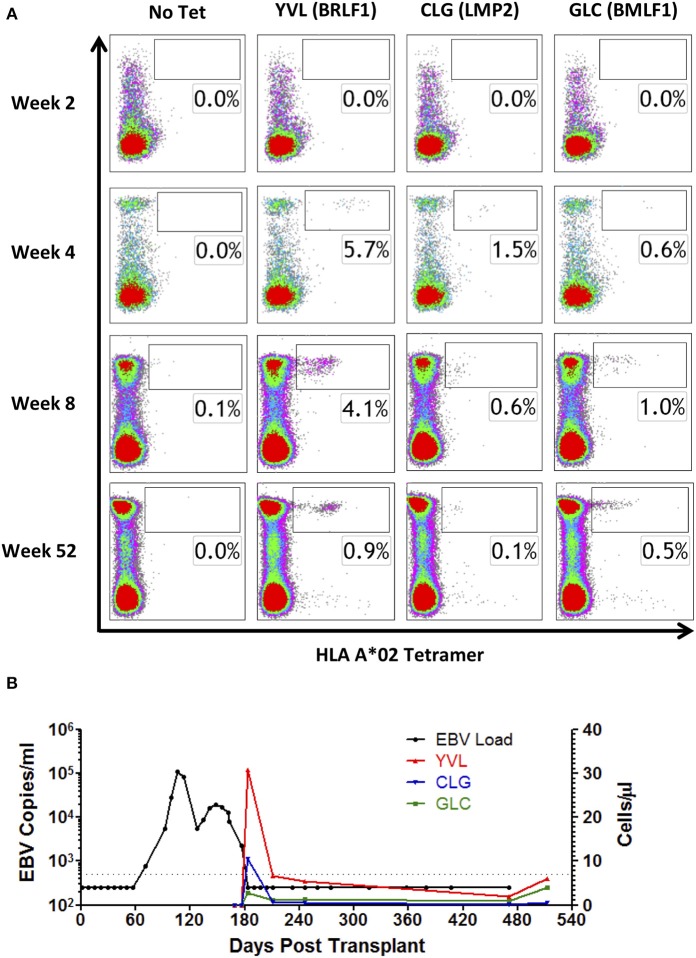
MHC I tetramer analysis of EBV epitope-specific CD8+ T-cell responses following DLI. PBMCs from HLA A^*^02-positive Patient A were stained with HLA A^*^02 tetramers containing YVL (BRLF1), CLG (LMP2), and GLC (BMLF1) epitope peptides. **(A)** Depicted results show lymphocytes co-stained for CD8+ and tetramer, compared to a no tetramer control (left column). Cell populations within the boxes indicate the percentage of tetramer-positive cells within the CD8+ T-cell population. **(B)** Comparison of response in whole blood viral load with the absolute frequency of EBV-specific T-cells recognizing YVL (BRLF1), CLG (LMP2), and GLC (BMLF1) in cells/μl over time. Vertical dotted lines represent the time of DLI.

Importantly, we also investigated the presence of the corresponding viral antigens within PTLD tumor tissue. As such, sections from the PTLD biopsy available from Patient A were subjected to immunohistochemistry for a range of EBV proteins (Figure 2C). This showed expression of EBNA-1, EBNA-2, LMP-1, and LMP-2 latent proteins, consistent with a growth transforming Latency III pattern of virus gene expression. Importantly, these included several proteins recognized by the T-cells expanding *in vivo* post-DLI (Figures 2, 3). Notably, the EBV lytic cycle antigen BZLF1, expression of which is responsible for initiating the virus replication program, was also readily detected in a small proportion of cells. However, late lytic cycle glycoprotein 350 (gp350), which contributes to the virus envelope and is one of the final proteins to be expressed from the EBV genome, was absent. Control gp350 staining of cells within the EBV-positive cell line Akata is shown in Figure S1.

### EBV-Specific T Cell Expansion Following DLI

As IFN-γ release may underestimate the total frequency of antigen-specific T-cells (36), we subsequently sought to quantify EBV-specific T-cells by flow cytometry, using MHC class I tetramers. Reagents were available for the HLA A^*^02 restricted EBV latent epitope CLG (LMP2) and the EBV lytic epitopes YVL (BRLF1) and GLC (BMLF1). These were used to analyze PBMCs from HLA A^*^02-positive Patient A, to determine responding T-cells as a proportion of circulating CD8+ T-cells (Figure 3A). Responses to all 3 tetramers were undetectable at 2 weeks following DLI, consistent with a general absence of CD8+ T-cells at this timepoint (Figure 1D). However, by 4 weeks after DLI, responses to YVL (BRLF1), and GLC (BMLF1) tetramers were markedly increased, comprising 5.7 and 1.5% of circulating CD8+ T-cells, respectively (Figure 3A). These responses were maximal at this timepoint and remained elevated at 8 weeks. The response to GLC (BMLF1) was less marked but also increased to a maximum of 1.0% of CD8+ T-cells at 8 weeks. Notably, 12 months after infusion of DLI the frequencies of MHC class I tetramer-specific cells within the CD8+ T-cell subset had fallen to levels typically found in a healthy EBV-seropositive adult (37). The frequency (cells/μl) of EBV tetramer-specific CD8+ T-cells was subsequently calculated (Figure 3B), revealing the full magnitude and kinetics of these responses. Importantly, this demonstrated the close alignment of EBV epitope-specific CD8+ T-cell expansion with resolution of viral DNAemia, particularly for the immunodominant EBV lytic peptide epitope YVL (BRLF1).

## Discussion

Whilst pre-emptive Rituximab constitutes an effective strategy to reduce PTLD-associated mortality after allo-HSCT, it nevertheless remains suboptimal. Treatment itself may confer an increased risk of opportunistic infection (38), and around 10% of patients treated pre-emptively develop established disease with Rituximab refractoriness (28). Given disappointing outcomes observed with cytotoxic chemotherapy, cellular therapies offer the best chance of rescue. However, effective responses to DLI or EBV CTLs are also not universal, and ~30% of patients fail treatment (18). Understanding the viral and immunological variables that influence outcome is crucial for the optimization of future adoptive immunotherapeutic approaches. In the current study the potential value of performing detailed immune characterization following administration of cellular therapy is exemplified with 2 cases of Rituximab-refractory PTLD successfully rescued with DLI. Here the administered cells contain the full repertoire of donor EBV immunity, providing an opportunity to investigate the antigen specificities of *in vivo* responding cells, unbiased by prior *in vitro* selection.

Both study patients received single doses of 1 × 10^6^/kg unselected transplant donor-derived lymphocytes from their EBV-immune stem cell donors. These infusions resulted in complete clinical response, comprising sustained resolution of EBV DNAemia and remission of radiological abnormalities. Notably, this was accompanied by marked expansion in circulating lymphocytes, with peak frequencies observed within 4 weeks of DLI. The lymphocyte expansions contained both CD8+ and CD4+ T-cells, consistent with previous reports that successful treatment of established PTLD correlates with restoration of T-cell numbers (18). Interestingly, we also observed simultaneous increases in CD3-CD56+ NK-cell numbers, potentially through stimulation by NKG2D, and/or DNAM-1 ligands upregulated on EBV-infected cells undergoing lytic replication (39, 40). Notably, *in vitro* studies have shown that this lymphocyte subset, some of which are primed for rapid IFN-γ production (41), may play a role in limiting B-cell transformation by EBV (42, 43). Whilst their contribution to the clinical responses seen against established PTLD following DLI is yet to be determined, increased NK frequencies have been reported in patients with controlled low-level EBV reactivation (44).

To dissect the CD8+ and CD4+ T-cell responses expanding *in vivo* after DLI, we performed detailed analysis of antigen specificities present both in the infused cell preparations, and in patient PBMCs collected pre- and post-DLI. Previous studies, undertaken in similarly treated patients, have been limited to estimations of global EBV-specific T-cell numbers using LCL or peptide pool stimulation followed by various *in vitro* readout assays (18, 45, 46). Whilst such methods provide a good measure of total EBV-specific responses, individual epitope specificities are not captured. In the current study, we instead used panels of patient HLA-relevant EBV epitope peptides to determine the frequency of individual epitope-specific responses by IFN-γ Elispot. This approach has previously been used to track the kinetics of infused peptide-selected T-cells of known specificity (23). In both patients, expanded T-cells contained immunodominant responses identical to those present in the DLI prior to infusion, i.e., YVL (BRLF1) for Patient A, and RPP (EBNA3A) for Patient B, and subdominant responses to a range of other CD8+ and CD4+ peptides. Crucially, detection of IFN-γ release in these *ex vivo* assays demonstrates that the expanded T-cells are able to function in response to their cognate antigen. Furthermore, we were also able to quantify absolute numbers of EBV epitope-specific CD8 T-cells for Patient A, whose HLA I type was amenable to analysis with HLA-A^*^02 restricted EBV peptide/MHC I tetramers. Unmistakably, the peak frequencies of tetramer positive cells in the blood coincided with the dynamics of total lymphocyte expansion, in line with reports of clinical responses occurring concurrently with expansion of LCL-reactive cells (18, 32). At this time, > 8% of all circulating CD8+ T-cells were specific for the available MHC I tetramers. Given that the observed responses represent only 3 epitopes from the entire virus genome, restricted through a single HLA I allele, it is likely that the majority of the expanded CD8+ T-cells are in fact EBV-specific.

In both cases, whilst immunodominant T-cell reactivity was preserved between DLI and patient, we observed notable differences in the repertoire of subdominant responses present in the DLI vs. those appearing *in vivo* after infusion. Thus, for Patient A the FLY (LMP2) peptide elicited the second largest response following infusion, whilst stronger responses in the DLI-EEN (EBNA3C) and FLD (BALF4)-were almost undetectable. Similarly, in Patient B reactivity against the CD8+ peptide pool, containing a number of lytic antigen-derived peptides, was more prominent 4 weeks post-infusion than in the DLI, whereas the TYS response of the DLI was undetectable after infusion. These differing patterns indicate non-uniform expansion of EBV epitope-specific T-cells present in the DLI following administration, and suggest that antigen encounter *in vivo* is shaping the observed responses. Furthermore, they may explain why attempts to simply correlate the dominant antigen specificities present in heterogeneous *in vitro*-stimulated therapeutic cell preparations with clinical outcome, have thus far proven unsuccessful (27, 46).

Immunohistochemistry on biopsy material from Patient A demonstrated expression of several proteins associated with the viral latency III program. Importantly, this included proteins that were targets of the expanded CD8+ T-cell response. Additionally, we detected unequivocal expression of the immediate early (IE) lytic cycle protein BZLF1. This transcription factor is the key initiator of EBV lytic cycle, and drives sequential expression of over 60 viral proteins involved in virus replication (3). However, despite widespread BZLF1 expression in the biopsy, we could not detect expression of the late (L) virion structural protein gp350. Such observations accord with earlier evidence that lytic cycle may not always progress to late stages in PTLD tumors (7, 8). In this regard, abortive lytic cycle has been suggested as a possible pathogenic mechanism, whereby IE and E viral protein expression might enhance tumourigenesis (47) without completing replication, which would otherwise eliminate the host cell (48). Interestingly, although we were only able to analyze a limited number of epitopes, this pattern was reflected in the T-cell specificities expanded *in vivo*. Thus, the immunodominant reactivity in the blood of Patient A following DLI was against the IE epitope YVL (BRLF1), whereas L epitope FLD (BALF4) responses were absent during the time of PTLD resolution. These data indicate that tumor antigen expression drives non-uniform T-cell expansion, and suggest that T-cells active against latent and early lytic antigens may be more therapeutically important than L antigen-specific responses in the setting of PTLD.

Collectively, this study demonstrates the importance of studying the antigen specificity of T-cell responses that expand *in vivo* following DLI. The application of this approach to a larger cohort of patients, with comparative analysis of individuals according to clinical response may deliver essential advances in the development of adoptive cell therapy for PTLD. Furthermore, extending the analysis of T-cell responses after DLI to include other viruses, such as Cytomegalovirus and Adenovirus will afford the opportunity to confirm that T-cell expansion is indeed driven by specific viral antigens. Of course, the study of virus-specific responses in recipients of DLI will not in itself inform the risk of potentially life-threatening acute GvHD, which is the principal limitation of DLI. However, it is notable that strategies, which do not require the prior selection of particular EBV antigen-specific T-cells by *ex vivo* purification and/or culture, might be used to mitigate this risk. For example, prior depletion of naïve T-cells from the DLI preparation has the potential to reduce the risk of GvHD, without compromising the range of EBV antigens targeted or EBV-specific cytotoxicity (49).

In summary, we report the successful treatment of Rituximab-refractory PTLD arising after allo-HSCT using DLI. Moreover, we have undertaken detailed analyses of virus-specific immune response developing *in vivo* following adoptive transfer. Disease resolution was found to coincide with marked expansion of lymphocytes, including functional EBV epitope-specific CD8+ and CD4+ T-cells exhibiting reactivity against a range of latent and IE lytic antigens, which were also expressed in tumor biopsy material. Importantly, although immunodominant T cell reactivity was preserved between DLI and patients following infusion, expansion of other DLI-derived antigen-specific T-cells was not uniform. This indicates that presentation of viral epitopes from the tumor may drive the *in vivo* immune response. These findings have important implications for the optimization of future adoptive immunotherapeutic strategies, including those involving *ex vivo* selected T-cells (23, 24) or genetically engineered T-cells. Thus, similar analyses of post-DLI expanded T-cells from larger series of patients, including non-responders, promise to facilitate the identification of essential T-cell responses required for therapeutic efficacy.

## Data Availability Statement

The datasets generated for this study are available on request to the corresponding author.

## Ethics Statement

The studies involving human participants were reviewed and approved by West Midlands - Coventry and Warwickshire Research Ethics Committee. The patients/participants provided their written informed consent to participate in this study.

## Author Contributions

DB performed laboratory work, interpreted the data, and co-wrote the manuscript. GR performed laboratory work. CH performed clinical data collection. EN performed laboratory work. SH interpreted radiological investigations. PM interpreted the data and co-wrote the manuscript. NR provided clinical management. CF provided clinical management, performed clinical data collection, and co-wrote the manuscript. HL performed laboratory work, interpreted the data, co-wrote the manuscript, and designed and oversaw the research.

### Conflict of Interest

The authors declare that the research was conducted in the absence of any commercial or financial relationships that could be construed as a potential conflict of interest.
