# Supplementary material for: Non-uniform in vivo Expansion of Epstein-Barr Virus-Specific T-Cells Following Donor Lymphocyte Infusion for Post-transplant Lymphoproliferative Disease
Source: Front Immunol. 2019 Oct 29;10:2489. doi: 10.3389/fimmu.2019.02489 (PMC6828838; doi:10.3389/fimmu.2019.02489)
Supplement: Supplementary file 1 [file Data_Sheet_1.PDF]

Table S1. Peptides used in IFN-γ Elispot assay for Patient A

Peptide	HLA restriction	Protein	aa coordinates	Lat/Lyt
VEI	B44:02	EBNA3B	657-666	Lat
EEN	B44:02	EBNA3C	281-290	Lat
KEH	B44:02	EBNA3C	335-343	Lat
CLG	A02:01	LMP2	426-434	Lat
FLY	A02:01	LMP2	356-364	Lat
LLW	A02:01	LMP2	329-337	Lat
YLL	A02:01	LMP1	125-133	Lat
YVL	A02:01	BRLF1	109-117	Lyt
GLC	A02:01	BMLF1	280-288	Lyt
FLD	A02:01	BALF4	276-284	Lyt
TSL*	DRB1 01:01	EBNA1	515-528	Lat
GPW*	DRB1 01:01	EBNA3A	780-799	Lat
MVF*	DRB1 05:01	EBNA1	563-577	Lat
EDL*	DRB1 05:01	EBNA3A	364-383	Lat
LDL**	DRB1 05:01	BLLF1	61-81	Lyt
TDA**	DRB1 05:01	BNRF1	1238-1252	Lyt
SNP*	DRB5 01:01	EBNA1	474-489	Lat
LEK**	DRB5 01:01	BXLF2	126-140	Lyt
SDD*	DQB1 06:02	EBNA3C	386-400	Lat

*CD4+ latent pool
**CD4+ lytic pool

Table S2. Peptides used in IFN-γ Elispot assay for Patient B

Peptide	HLA restriction	Protein	aa coordinates	Lat/Lyt
RYS	A24:01	EBNA3A	246-253	Lat
TYS	A24:01	EBNA3B	217-225	Lat
TYG	A24:01	LMP2	419-427	Lat
RPGK	B07:02	EBNA1	72-80	Lat
IPQ	B07:02	EBNA1	528-536	Lat
RPP	B07:02	EBNA3A	379-387	Lat
QPR	B07:02	EBNA3C	881-889	Lat
YPR*	B07:02	BNRF1	1247-1257	Lyt
TPS*	B07:02	BFRF3	127-137	Lyt
SRL*	B14:01	BMLF1	435-444	Lyt
LQH*	Cw08	BZLF1	101-115	Lyt
VYG	DRB1 07:01	EBNA1	509-528	Lat
PRS	DRB1 07:01	EBNA2	276-295	Lat
SRD**	DRB1 07:01	BaRF1	185-199	Lyt
MVF	DRB1 15:01	EBNA1	563-577	Lat
EDL	DRB1 15:01	EBNA3A	364-383	Lat
LDL**	DRB1 15:01	BLLF1	61-81	Lyt
TDA**	DRB1 15:01	BNRF1	1238-1252	Lyt
SNP	DRB5 01:01	EBNA1	474-489	Lat
LEK**	DRB5 01:01	BXLF2	126-140	Lyt
ILR**	DRB4 01:01	EBNA3C	771-790	Lat

*CD8+ pool
**CD4+ pool

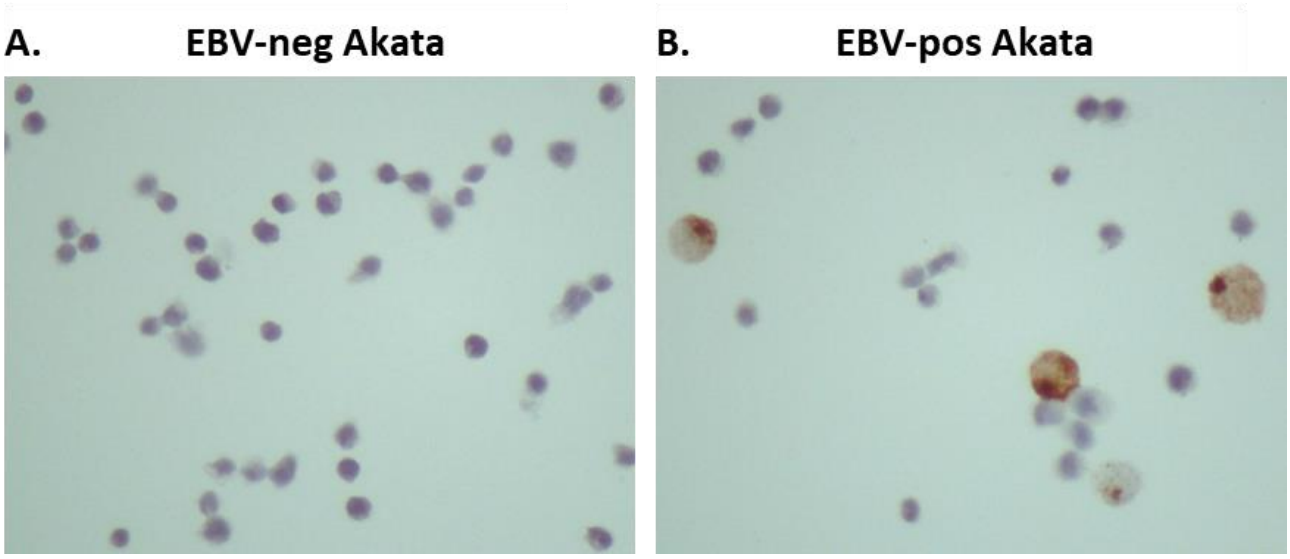

Figure S1. Gp350 expression in control cell lines. Gp350 staining on cytospins of (A) EBV-negative and (B) EBV-positive Akata cell lines. Images are magnified 40X.
